# De Novo Gastroesophageal Reflux Disease Symptoms Are Infrequent after Sleeve Gastrectomy at 2-Year Follow-Up Using a Comprehensive Preoperative Esophageal Assessment

**DOI:** 10.3390/jcm13020545

**Published:** 2024-01-18

**Authors:** Salvatore Tolone, Giovanni Conzo, Luigi Flagiello, Claudio Gambardella, Francesco Saverio Lucido, Luigi Brusciano, Simona Parisi, Nicola De Bortoli, Edoardo Vincenzo Savarino, Gianmattia Del Genio, Ludovico Docimo

**Affiliations:** 1 Division of General, Mininvasive, Oncologic and Bariatric Surgery, Department of Advanced Medical and Surgical Sciences, University of Campania “Luigi Vanvitelli”, Via Pansini 5, 80131 Naples, Italyluigi.flagiello1@studenti.unicampania.it (L.F.); luigi.brusciano@unicampania.it (L.B.); simona.parisi@unicampania.it (S.P.); gianmattia.delgenio@unicampania.it (G.D.G.); ludovico.docimo@unicampania.it (L.D.); 2 Division of Gastroenterology, Department of Translational Research and New Technologies in Medicine and Surgery, University of Pisa, 56127 Pisa, Italy; nicola.debortoli@unipi.it; 3 Division of Gastroenterology, Department of Surgical, Oncological and Gastroenterological Sciences, University of Padua, 35128 Padua, Italy; edoardo.savarino@unipd.it

**Keywords:** obesity, GERD, sleeve gastrectomy

## Abstract

Pathological obesity is a growing public health concern, and its association with gastroesophageal reflux disease (GERD) poses challenges in selecting the appropriate bariatric procedure. Sleeve gastrectomy (SG) has become a popular choice due to its simplicity and effectiveness in weight loss. However, concerns regarding postoperative GERD have been raised. This study aimed to evaluate the association between preoperative assessment of esophageal function and the risk of developing postoperative GERD in patients undergoing SG. A comprehensive evaluation was conducted, including symptom assessment, upper endoscopy, high-resolution esophageal manometry (HRM), and 24 h esophageal pH impedance monitoring (MII-pH). A total of 500 obese patients were included, and their data were compared with 25 healthy volunteers. This study revealed that patients without GERD symptoms, normal endoscopy, HRM, and MII-pH were suitable candidates for SG, with low risk of developing postoperative GERD. The addition of fundoplication techniques to SG may be considered in patients with mild reflux or those at risk of developing it. This study emphasizes the importance of preoperative evaluation in selecting the appropriate bariatric procedure to minimize the risk of postoperative GERD and expand the indications for SG in obese patients.

## 1. Introduction

Pathological obesity is a growing public health emergency in Western countries, with over 30% of European adults being overweight and approximately 10% of the population affected by class III obesity [1]. This condition is strongly associated with gastroesophageal reflux disease (GERD) due to multiple pathophysiological mechanisms such as increased intragastric and intra-abdominal pressure, altered inspiratory pressure, changes in chemical clearance in the esophagus, and a high incidence of hiatal hernia. Surgeons must consider this association when selecting the appropriate bariatric procedure, which should ensure effective and long-lasting weight loss and improve or resolve reflux. Sleeve gastrectomy (SG) has become the most commonly used bariatric surgery procedure worldwide due to its technical simplicity, reduced operative time, rapid weight loss induction, and fewer late complications [2]. However, like any gastric anatomy-modifying surgery, SG impacts the functionality of both the stomach and the esophagus, potentially leading to postoperative “de novo” reflux [3]. Recent studies have reported an increased incidence of GERD and Barrett’s esophagus after SG, particularly associated with duodenal–gastric bile reflux, leading some bariatric surgeons to consider hiatal hernia or GERD as a relative contraindication for SG in obese patients [4]. To address this issue, a surgical technique involving the addition of an anterior Dor fundoplication to conventional SG (D-Sleeve) or a Nissen fundoplication has been developed to limit postoperative GERD risk and extend SG indications to patients with mild reflux or those at risk of developing it [5,6,7]. Preoperative evaluation, including upper gastrointestinal endoscopy, pH impedance monitoring (MII-pH), and high-resolution esophageal manometry (HRM), is essential to correctly diagnose GERD presence and thus could be useful in selecting appropriate candidates for this technique. The aim of our study was to verify if the indication for SG provided after a comprehensive assessment of esophageal function (by the means of symptoms evaluation, upper endoscopy, HRM, and MII-pH monitoring) was associated with low risk of developing postoperative GERD.

## 2. Materials and Methods

This study included 500 obese patients (280 males, 220 females) with a BMI ≥ 35 who were candidates for bariatric surgery at a Department of General, Minimally Invasive, Oncological, and Obesity Surgery from 2014 to 2021, according to Italian guidelines. The patients underwent a standardized protocol to diagnose the presence of gastroesophageal reflux, which included a clinical evaluation, upper endoscopy (UE), high-resolution esophageal manometry (HRM), and 24 h esophageal MII-pH monitoring (MII-pH). Patients who had already undergone gastric surgery or had a large hiatal hernia (defined as greater than 3 cm at HRM or on UE) were excluded. This study also collected data from 28 healthy volunteers (HV) with a BMI of 20–25, negative endoscopy, normal MII-pH and HRM, not matched for age and sex with the study population. The protocol was approved by the IRB, and each patient signed an informed consent form after being clearly informed. Anthropometric data such as weight, height, and BMI were collected for all patients. Each study subject responded to a questionnaire to evaluate symptoms such as heartburn, regurgitation, dysphagia, and retrosternal pain, useful for the diagnosis of GERD (GerdQ score) (Figure S1) [8], with questions related to the possible presence of symptoms in the last seven days and their frequency (days 0, 1, 2–3, 4–7). Patients who reported heartburn and regurgitation were classified as likely affected by GERD (SYMPTOMS+), while those who did not report such symptoms were classified as SYMPTOMS−. The presence of esophagitis was evaluated according to the Los Angeles classification, while in the presence of hiatal hernia, the Hill classification was followed [9]. Subjects with a small hiatal hernia (1–2 cm) and/or grade A esophagitis were defined as likely positive for GERD (UE+); all the patients SYMPTOMS+ and those UE+ (even if SYMPTOMS−) underwent HRM and MII-pH monitoring.

All patients underwent high-resolution manometry, using a solid manometric catheter with 32 sensors, combined with an impedance recording system consisting of 36 circular and unidirectional sensors sensitive to pressure spaced at intervals of 1 cm and 9 impedance recording rings (5 impedance segments) spaced at intervals of 1 cm (Sandhill Scientific Inc., Highlands Ranch, CO, USA). Data control and analysis were performed with dedicated software (Sandhill Bioview, Sandhill Scientific) after appropriate thermal compensation. The candidates did not take any medication and remained fasting for at least 6 h before undergoing manometry. The examination was performed with the patients in the supine position. The catheter was introduced transnasally and positioned to record the entire esophageal length with at least 5 intragastric sensors to better study the esophagus and the esophagogastric junction (EGJ). The HRM protocol, according to the Chicago Classification v3.0, included a 5 min period to evaluate the pressure of the esophagogastric junction (EGJ) at rest, followed by 10 swallows of 5 mL of 0.3% saline solution to evaluate the function of the esophagus in its entirety. The Chicago classification v3.0 was used to classify the esophageal motility disorders. The classification system includes three major categories: esophageal motility disorders, esophagogastric junction outflow obstruction, and major disorders of peristalsis. Each category is further divided into subcategories based on the specific motility patterns observed on HRM [10,11,12]. According to this protocol, lower esophageal sphincter pressure (LESp), esophago-gastric junction (EGJ) type (I if LES and crura were overlapping, II when an axial separation was 1–2 cm, III when the axial separation was greater than 2 cm), EGJ contractile integral (EGJ-CI, defined during 3 respiratory acts as cm*sec*mmHg at EGJ), gastroesophageal pressure gradient (GEPG, as the difference in basal pressure between esophageal lumen and gastric lumen), and motility pattern were assessed.

Ambulatory 24 h studies with intraluminal pH and impedance sensors were conducted to document the presence of GERD. The data obtained from the catheters were processed using dedicated software, and several parameters were studied, including acid exposure time, abnormal AET%, total number of reflux episodes and their quality (acidic, weakly acidic, and weakly alkaline), symptom association index, and probability of symptom association. GERD presence was re-defined according to the Lyon Consensus. Inconclusive GERD was then evaluated as conclusive when at least 1 adjunctive parameter at HRM and MII-pH was found [13,14,15].

Patients with absence of any sign of GERD (Symptoms-, normal UE, normal HRM, and normal MII-pH) were submitted to SG. The remaining patients were excluded from this study and underwent Dor-Sleeve, Nissen-Sleeve, Toupet-Sleeve or Roux-en-Y Gastric bypass.

In all patients, sleeve gastrectomy was conducted using a 4-trocar technique. To provide a succinct overview, the procedure involved initiating dissection of the gastric greater curvature at a point 4 to 6 cm distal to the pylorus and extending it 1 cm beyond the His angle. This specific approach was chosen to steer clear of the critical zone associated with reduced blood supply. The sleeve gastrectomy (LSG) itself was performed using the Echelon Endoscopic Stapler (J&J), employing cartridges of various colors (green, gold, and blue) depending on the thickness of the stomach. A bougie with a diameter of 42 Fr was utilized during surgery. In all patients, additional overrunning suture was applied following the conventional SG procedure. This reinforcement consisted of a single overrunning suture using a barbed suture to secure the sero-serosal layers of the stomach, thereby ensuring complete invagination of the staple line. Subsequently, a double intraoperative leak test was conducted in all cases. This involved insufflating air via intraoperative endoscopy while submerging the sleeved stomach in a sterile solution. Additionally, a conventional methylene blue test was administered. Along the staple line, a drainage tube was inserted.

On the fifth day post operation, an X-ray using a hydro-soluble contrast agent (Gastrographin) was performed. The purpose of this examination was to identify any potential leaks.

Descriptive statistics were used to summarize the data. Continuous variables were expressed as mean ± standard deviation or median (interquartile range), and categorical variables were expressed as frequency and percentage. The chi-square test or Fisher’s exact test was used to compare categorical variables, and Student’s *t*-test or the Mann–Whitney U test was used to compare continuous variables. A *p*-value of less than 0.05 was considered statistically significant. All statistical analyses were performed using SPSS version 23.0 (IBM Corp., Armonk, NY, USA).

## 3. Results

A total of 500 patients (280 males, 56%, and 220 females, 44%) were studied. The mean age of the patients was 36 years (SD ± 14.8), while the mean height and weight were 167.3 cm (SD ± 9.2) and 132.1 kg (SD ± 26.8), respectively. The mean BMI of the patients was 44.2 kg/m^2^ (SD ± 7.0). There were no significant differences between males and females in terms of BMI. The HV group included 12 males and 16 females, mean age 25 years (SD ± 5.2), mean BMI 22 (SD ± 0.8). Using the GerdQ for the evaluation of symptoms attributable to reflux disease, 410 patients tested positive (SYMPTOMS+ 82%), while 90 patients did not report any symptoms (SYMPTOMS− 18%).

In 260 patients (52%), at UE, the presence of hiatal hernia of any grade was reported. Of these, 35% (90 patients) had a grade IV hiatal hernia according to Hill’s classification. These patients were informed of this pathological condition and were either candidates for malabsorptive/mixed bariatric surgery or directed towards a possible surgical intervention on the gastroesophageal junction before bariatric surgery. The remaining 170 patients had a grade II–III hiatal hernia according to Hill’s classification (cardiac incontinence). In 70 patients, Los Angeles grade A and B esophagitis was detected (40 and 30 patients, respectively). In accordance with the endoscopic and clinical evaluation results, our population was stratified into three groups as follows: group 1 (S−.UE−) consisted of 89 patients (17.8%) negative for symptoms and endoscopy, group 2 (S+.UE−) consisted of 149 patients (29.8%) positive for symptoms but negative on UE, and group 3 (S+.UE+) consisted of 262 patients (52.4%) positive for both.

High-resolution manometry revealed that intragastric pressure was significantly higher in obese patients compared to the HV group during both inspiration and expiration (16 mmHg ± 2 vs. 6 ± 1.5, *p* < 0.001). There were no significant differences in intragastric pressure values among the three groups. Similar results were observed for intraesophageal pressure, which was significantly higher in obese patients compared to the HV group, without substantial differences among the three groups. Mean GEPG during inspiration and expiration were significantly higher in obese patients compared to normal subjects (10.2 mmHg ± 3 vs. 4 ± 2, *p* < 0.05). The spatial separation between the lower esophageal sphincter (LES) and the diaphragmatic crus was increased in obese patients compared to the HV group; however, only in group 3 was the spatial separation between LES and CD significantly increased (presence of hiatal hernia in 262/262 patients). The study of peristalsis showed only ineffective motility patterns, without any specific motor disorders. In particular, group 1 did not present any difference in the incidence of weak peristalsis compared to the HV group (78.9% vs. 82%, respectively); conversely, group 2 and group 3 showed a significant increase in weak peristalsis (69% and 40%, respectively). Regarding the 24 h MII-pH, group 1 reported an increase in acid exposure time (AET) compared to the HV group. Groups 2 and 3 presented a significant increase in acid exposure time (in both supine and upright positions), which was pathological in all subjects. In particular, group 3 showed a significant increase in supine acid exposure time compared to group 2. Moreover, group 3 presented a higher number of reflux episodes (acidic and weakly acidic) compared to the HV, group 1, and group 2.

Group 1 (89 patients) tested negative for GERD and underwent SG. All surgical procedures were uneventful. Two patients (2.5%) were reoperated on post-operative day 1 due to bleeding from gastroepiploic vessels. No leakage was detected. Eleven patients were lost at FU. The excess weight loss (EWL) at 6-month, 1-year, and 2-year FU in 78 patients was 48%, 64%, 63%, respectively.

Two patients (2.5%) reported de novo GERD symptom development. At X-ray, both of them had middle gastric stricture and GERD symptoms relieved after endoscopic dilatation. Twelve patients assumed PPIs for gastritis, but not for symptoms related to GERD. Post-operative UE was available in 43 patients, and in none of them was esophagitis or Barrett’s esophagus present.

## 4. Discussion

Sleeve gastrectomy involves the removal of a significant portion of the stomach, leaving a narrow tube-shaped stomach. The reduction in the stomach’s size and the decreased secretion of ghrelin hormone have been associated with a reduction in GERD symptoms. However, some studies have reported an increased incidence of de novo GERD symptoms after sleeve gastrectomy. A systematic review and meta-analysis of 22 studies reported an increased incidence of de novo GERD symptoms after sleeve gastrectomy, with a pooled incidence of 23.6% (95% CI 17.3–30.9) [16]. Another systematic review and meta-analysis of 29 studies reported an incidence of 14.4% (95% CI 9.9–19.8) [17]. The reason for the discrepancy in the incidence rate could be due to differences in patient selection, surgical technique, and follow-up duration. GERD is the most significant risk factor for the development of Barrett’s esophagus. The effect of sleeve gastrectomy on Barrett’s esophagus is still unclear. Some studies have reported a decreased incidence of Barrett’s esophagus after sleeve gastrectomy. A retrospective study of 2074 patients who underwent bariatric surgery reported a decreased incidence of Barrett’s esophagus after sleeve gastrectomy [18]. However, other studies have reported an increased incidence of Barrett’s esophagus after sleeve gastrectomy [19]. A systematic review and meta-analysis of 15 studies reported a pooled incidence of 2.7% (95% CI 1.0–5.0) of Barrett’s esophagus after sleeve gastrectomy [20].

Some studies have reported a reduction in GERD symptoms after sleeve gastrectomy. The reason for the improvement in GERD symptoms could be due to the reduction in the stomach’s size and the decreased secretion of ghrelin hormone [21]. Also, weight loss can play a role in the decrease in GERD symptoms and frequency; this may support the data we found.

Upper endoscopy, high-resolution manometry, and pH impedance monitoring play a crucial role in the correct diagnosis of GERD in an obese patient and guide the selection of the appropriate bariatric procedure [22,23,24,25,26].

The above-described preoperative study led to the formulation of a decision algorithm that allowed the selection of sleeve gastrectomy as a therapeutic strategy in patients without increasing the risk of developing postoperative de novo GERD [27]. This decision algorithm involves, in the first instance, the evaluation of reflux-related symptoms using GerdQ and the execution of upper endoscopy, a diagnostic examination that obese patients undergoing bariatric surgery should undergo according to international guidelines. If UE is substantially normal and there is no reflux-related symptomatology (negative GerdQ), the patient can be a candidate for sleeve gastrectomy, as confirmed by HRM and MII-pH. In fact, our cohort of patients showed very low incidence of de novo GERD, and the observed incidence was due to an anatomical complication of SG [28,29,30,31].

On the other hand, testing obese patients with GerdQ, UE, HRM, and MII-pH can also offer the possibility of selecting patients for an SG combined with a fundoplication or submission to Roux-en-Y gastric bypass, reducing the consequences of de novo GERD and Barrett’s esophagus. Several studies have reported a significant improvement in GERD symptoms following RYGB. The mechanism behind this improvement is thought to be multifactorial. RYGB involves the creation of a small gastric pouch, which limits the capacity of the stomach and reduces pressure on the lower esophageal sphincter. Additionally, the procedure reroutes the digestive tract, diverting gastric acid and bile away from the esophagus.

Long-term studies have consistently shown a sustained reduction in GERD symptoms post RYGB. A prospective cohort study by Csendes et al. [32] demonstrated that 90% of patients experienced complete resolution of GERD symptoms five years after RYGB. These findings are further supported by a meta-analysis conducted by Tie et al., which revealed a significant decrease in GERD symptoms and esophagitis after RYGB compared to non-surgical interventions [33].

One limitation of this study is represented by the fact that follow-up was performed only in part by means of UE, so we may have missed some patients with asymptomatic GERD. However, we previously reported a study with comprehensive assessment both in pre- and in post- operative time in a small cohort of patients, supporting these results [5]. Also, a 5-year follow-up is needed in order to compare the present data with other studies performed with the same range of time. Another limitation may be the strict inclusive criteria we used. For instance, we excluded large hiatal hernia (>3 cm) in order to have a more homogeneous cohort, since large hiatal hernia is a confounder for esophageal symptoms and for the need for surgical repair.

## 5. Conclusions

Sleeve gastrectomy is an effective bariatric procedure for the treatment of obesity and its associated comorbidities. However, its effect on GERD is still debated. Some studies have reported an increased incidence of de novo GERD symptoms after sleeve gastrectomy, while others have reported a reduction in GERD symptoms. The effect of sleeve gastrectomy on Barrett’s esophagus is still unclear. Instrumental tests such as upper endoscopy, high-resolution manometry, and pH impedance monitoring play a crucial role in the correct diagnosis of GERD in an obese patient and guide the selection of the appropriate bariatric procedure. Further studies are needed to determine the long-term effect of sleeve gastrectomy on GERD and Barrett’s esophagus.

## Data Availability

Data are contained within the article.

## Supplementary Materials

The following supporting information can be downloaded at: https://www.mdpi.com/article/10.3390/jcm13020545/s1, Figure S1: GERDQ self assessment questionnaire.
