# Supplementary material for: De Novo Gastroesophageal Reflux Disease Symptoms Are Infrequent after Sleeve Gastrectomy at 2-Year Follow-Up Using a Comprehensive Preoperative Esophageal Assessment
Source: J Clin Med. 2024 Jan 18;13(2):545. doi: 10.3390/jcm13020545 (PMC10816106; doi:10.3390/jcm13020545)
Supplement: Supplementary file 1 [file jcm-13-00545-s001.zip › jcm-2440292-supplementary.pdf]

Journal of
Clinical Medicine

MDPI

	Symptoms in the previous week	Symptom presence			
		0 days	1 day	2-3 days	4-7 days
	**Question:**				
1.	How often did you have a burning feeling behind your breastbone (heartburn)?	0	1	2	3
2.	How often did you have stomach contents (liquid or food) moving upwards to your throat or mouth (regurgitation)?	0	1	2	3
3.	How often did you have a pain in the center of the upper stomach?	3	2	1	0
4.	How often did you have nausea?	3	2	1	0
5.	How often did you have difficulty getting a good night's sleep because of your heartburn and/or regurgitation?	0	1	2	3
6.	How often did you take additional medication for your heartburn and/or regurgitation other than what the physician told you to take (such as Maalox)?	0	1	2	3

Figure S1. GERDQ self assessment questionnaire.
